# Annotations

**Published:** 1910-12-03

**Authors:** 


### ANNOTATIONS.

### The Consumption Crusade.

The National Association for the Prevention of Consumption and other Forms of Tuberculosis will command the heartiest sympathy of the medical profession in the great poster campaign which it has recently inaugurated. Throughout the United Kingdom hoardings will display posters which state some of the statistics of the ravages of this preventable disease; and it is to be trusted that the appeal for further funds, which the same posters convey, will receive an encouraging response. The venture has the approval of the leaders of both political parties, who have thus found at any rate one subject which is beyond the wrangling of parties. On previous occasions approval has been expressed in these columns of the endeavours which this society is making to educate the people to a knowledge of the vast importance of eradicating tuberculosis; and in renewing the appeal to the profession to support it by their influence and example, it may be pointed out that the work of the travelling caravans, which have already done so much good, is sadly hampered for lack of funds. The Tuberculosis Exhibition also, which was a splendid success in Whitechapel and other poor districts of London, and in the provincial towns where it has been opened, is capable of greatly multiplying its usefulness if financial restrictions can be eliminated; a continuous tour of the country could and would be undertaken if circumstances permitted. In the purely educational work to which this exhibition is devoted there is a grand opportunity for the profession to co-operate, not only by driving home to their patients the truth and logic of the anti-tuberculosis campaign, but by soliciting from their more affluent patients subscriptions to enable its activities to be greatly extended.

### Smallpox in South America.

The awakening and industrial progress of the South American Republics is one of the most considerable features of the world's economic progress in recent years. It is therefore not astonishing to find that public health is receiving attention in the most progressive and civilised of these great territories: the Instituto de Oswaldo Cruz, for instance, publishes research work which is quoted widely in Europe. It is evident that the smallpox problem is to be tackled in earnest at Montevideo, Uruguay. The ravages of this disease have been much curtailed there, as might be expected, by improved sanitation during recent years. But a limit seems to have been reached beyond which further improvement is necessary, for the number of cases, which diminished steadily for some years, is now again increasing and will reach this year well over a thousand, with a mortality of 35 per cent. It has been proved that in the common lodging-houses, where vaccination is compulsory, there are one-thirteenth, proportionally, of the cases which occur among the rest of the population, among whom vaccination is optional; yet these lucky lodgers are in much worse circumstances otherwise for the acquisition of the disease. The Government is therefore introducing a Bill the gist of which is as follows: Vaccination and revaccination against smallpox are compulsory for all. Animal lymph, provided by Government, shall be exclusively used. Every public official, even down to the army and police, must be both vaccinated and revaccinated. Violation of the law is punishable with one day's imprisonment, or a fine of four dollars, *every month* until the obligation is fulfilled. Parents and guardians are responsible for the vaccination of minors. Fines collected shall go towards the expenses caused by the Act, under which six public vaccinators are to be appointed. How long will England lag behind even South America?

### Mr. Schroder's Deputy.

Not long ago a protest was offered in these columns (The Hospital, November 5, 1910, p. 155) against the injudicious action of the London County Council in overruling its own General Purposes Committee in the matter of the Central London Coronership appointment. For reasons then given, and on broad grounds of public interest and policy, the selection of Mr. Schröder for this post and the rejection of the Committee's nominee (Mr. Oddie) were described as unsatisfactory, and an encouragement of the very lax standard of duty which the late Mr. Danford Thomas saw fit to adopt. Within the few weeks that have since elapsed the most striking proof has been offered of the justice of that contention. The daily Press of November 26 contains a curt announcement that Mr. F. Danford Thomas, barrister-at-law, has been appointed by the Coroner for Central London to act as his deputy! Mr. Thomas may have, for all we know, many qualifications for this post, but he lacks that which is most essential of all, in that he is not a medical man. The real point is still (as with the appointment of Mr. Schröder) not a personal one at all. However eligible Mr. Danford Thomas, junior, may be—even if he were a medical man as well as a lawyer—an opportunity is still afforded for suspicious persons to raise a cry of favouritism, thus to appoint him in the very district where his relative allowed 9,000 inquests to be done by the very deputy who is now Coroner. The notion, whether it be well founded or not, that family connections are a surer passport than merit, experience, or qualifications to employment as a Coroner is bound to be fostered by this act of Mr. Schröder's; and it is to be feared that the natural result will be to discourage still further those who have had ambitions to fit themselves for this work by qualifying themselves both in medicine and the law. Mr. Danford Thomas is reported in the newspapers as conducting an inquest on November 29, so it is evident that Mr. Schröder is losing little time in imitating the system under which he himself deputised so long; hence our allusion to the subject.

